# Clinical Evaluation of a Newly Developed Guidewire for Pancreatobiliary Endoscopy

**DOI:** 10.3390/jcm9124059

**Published:** 2020-12-16

**Authors:** Shigeto Ishii, Toshio Fujisawa, Hiroyuki Isayama, Shingo Asahara, Shingo Ogiwara, Hironao Okubo, Hisafumi Yamagata, Mako Ushio, Sho Takahashi, Hiroki Okawa, Wataru Yamagata, Yoshihiro Okawa, Akinori Suzuki, Yusuke Takasaki, Kazushige Ochiai, Ko Tomishima, Hiroaki Saito, Shuichiro Shiina, Takaaki Ikari

**Affiliations:** 1 Department of Gastroenterology, Graduate School of Medicine, Juntendo University, Tokyo 113-8421, Japan; sishii@juntendo.ac.jp (S.I.); t-fujisawa@juntendo.ac.jp (T.F.); m-ushio@juntendo.ac.jp (M.U.); sho-takahashi@juntendo.ac.jp (S.T.); wataru.yamagata@gmail.com (W.Y.); y.okawa.kl@juntendo.ac.jp (Y.O.); suzukia@juntendo.ac.jp (A.S.); ytakasa@juntendo.ac.jp (Y.T.); k.ochiai.qd@juntendo.ac.jp (K.O.); tomishim@juntendo.ac.jp (K.T.); sshiina@juntendo.ac.jp (S.S.); 2 Department of Gastroenterology, Chiba Tokushukai Hospital, Chiba 274-8505, Japan; s.asahara@chibatoku.or.jp; 3 Department of Gastroenterology, Juntendo University School of Medicine Urayasu Hospital, Chiba 279-0021, Japan; s-ogiwara@juntendo-urayasu.jp (S.O.); med20618@juntendo-urayasu.jp (H.O.); 4 Department of Gastroenterology, Juntendo University School of Medicine Nerima Hospital, Tokyo 117-8521, Japan; drokubo@juntendo-nerima.jp (H.O.); hiloaki@juntendo.ac.jp (H.S.); 5 Department of Gastroenterology, Tobu Chiiki Hospital, Tokyo 125-8512, Japan; hyamaga@juntendo.ac.jp (H.Y.); takaakiikaripanc@gmail.com (T.I.)

**Keywords:** guidewire, common bile duct stone, malignant biliary obstruction, acute cholecystitis

## Abstract

Background: The guidewire (GW) plays an important role in pancreatobiliary endoscopy. GW quality is a critical factor in the effectiveness and efficiency of pancreatobiliary endoscopy. In this study, we evaluate a new 0.025 inch multipurpose endoscopic GW: the M-Through. Methods: Our study was a multicenter retrospective analysis. We enrolled patients who underwent endoscopic procedures using the M-Through between May 2018 and April 2020. Patients receiving the following endoscopic treatments were enrolled: common bile duct (CBD) stone extraction, endoscopic drainage for distal and hilar malignant biliary obstruction (MBO), and endoscopic drainage for acute cholecystitis. For each procedure, we examined the rate of success without GW exchange. Results: A total of 170 patients (80 with CBD stones, 60 with MBO, and 30 with cholecystitis) were enrolled. The rate of completion without GW exchange was 100% for CBD stone extraction, 83.3% for endoscopic drainage for MBO, and 43.3% for endoscopic drainage for cholecystitis. In unsuccessful cholecystitis cases with the original GW manipulator, 1 of 8 cases succeeded in the manipulator exchange. Including 6 cases who changed GW after the manipulator exchange, 11 of 16 cases succeeded in changing GW. There was significant difference in the success rate between the manipulator exchange and GW exchange (*p* = 0.03). The insertion of devices and stent placement after biliary cannulation (regardless of type) were almost completed with M-through. We observed no intraoperative GW-related adverse events such as perforation and bleeding due to manipulation. Conclusion: The 0.025 inch M-Through can be used for endoscopic retrograde cholangiopancreatography-related procedures efficiently and safely. Our study found high rates of success without GW exchange in all procedures except for endoscopic drainage for cholecystitis. This GW is considered (1) excellent for supportability of device insertion to remove CBD stones; (2) good for seeking the biliary malignant stricture but sometimes need the help of a hydrophilic GW; (3) suboptimal for gallbladder drainage that require a high level of seeking ability.

## 1. Introduction

Endoscopy has gained widespread international acceptance as a treatment for various pancreatobiliary diseases. GW quality is a critical factor in the effectiveness and efficiency of pancreatobiliary endoscopy. GWs have many uses: guiding catheters, supporting device insertion, exchanging devices, entering the branches of a duct, and passing through strictures. GWs were originally made only of stainless-steel wire, with spring coils used to increase flexibility. However, this type of GW was not useful for passing through strictures and entering branches of the pancreatic and bile ducts, only for exchanging devices. A hydrophilic GW (Radifocus M; Terumo Corporation, Tokyo, Japan), by contrast, is good for passing through strictures and entering branches but less useful for device exchange. It is therefore common for a procedure to require two GWs, especially for cases with strictures.

The first GW dedicated to endoscopic procedures was the “Zebra Wire” (Boston Scientific Corporation, Marlborough, MA, USA). This wire was plastic-coated but had limited ability to pass through strictures and selected branches. Plastic-coated GWs have less friction than coiled spring wires and are very useful in device exchange. The “Jagwire” (Boston Scientific Corporation) was the first multipurpose GW with a hydrophilic tip, but its ability to pass through strictures and enter side branches is poor compared to that of a hydrophilic GW. However, the introduction of the Jagwire allowed us to perform many endoscopic procedures with a single GW.

For a long time, the standard diameter of a GW was 0.035 inches. However, the use of 0.025 inch GWs has gradually increased. Because of large differences between the diameters of GWs and devices, thinner GWs may simplify device exchange and make it easier to rotate a GW within a device. A thin GW with a hydrophilic tip has a greater ability to advance through strictures and enter branches. A soft flexible tip protects the pancreas and biliary mucosa from injury. Ease of device exchange, which depends on guidewire stiffness, was a problem with thin GWs. However, modern 0.025 inch GWs are stiff enough for many procedures [1,2,3,4].

Assessing the clinical value of GWs is difficult because few studies have evaluated GWs [1]. A small number of bench tests comparing GW features have been conducted [5]. There have been some publications on the results of wire-guided cannulation using newly developed GWs [6,7,8,9].

We recently had the opportunity to evaluate a new 0.025 inch endoscopic GW: the M-Through (Asahi Intecc Corp., Aichi, Japan). This GW has a hydrophilic tip and high torquability. It is characterized as a multipurpose GW that is good at passing through strictures and entering branches. We sought to determine the value of this multipurpose GW in clinical practice and assess whether it was possible to complete procedures without changing the GW. Thus, the purpose of the present study was to determine the rate of success without GW exchange in selected procedures.

## 2. Patients and Methods

### 2.1. Study Design

Our study was a multicenter retrospective analysis. Three academic centers and two referral centers participated in the study. Since the start of this study, we have basically used M-through for consecutive cases. The contents to be examined were set in advance, and the data of each case was examined retrospectively. The study protocol was approved by the institutional review board of each institution. Participants provided informed consent for the procedures performed. Informed consent to participate in the study was obtained in the form of an opt-out on the website of Juntendo University. The procedures were performed by expert endoscopists with experience conducting more than 100 endoscopic retrograde cholangiopancreatography (ERCP)-related procedures each year, or by trainees working under the supervision of expert endoscopists.

### 2.2. Inclusion Criteria

We enrolled patients who underwent endoscopic procedures using the M-Through between May 2018 and April 2020. Patients receiving the following endoscopic treatments were enrolled: common bile duct (CBD) stone extraction, endoscopic drainage for distal and hilar malignant biliary obstruction (MBO), and endoscopic drainage for acute cholecystitis.

### 2.3. Endoscopic Procedures with the M-Through GW

**Figure 1:**
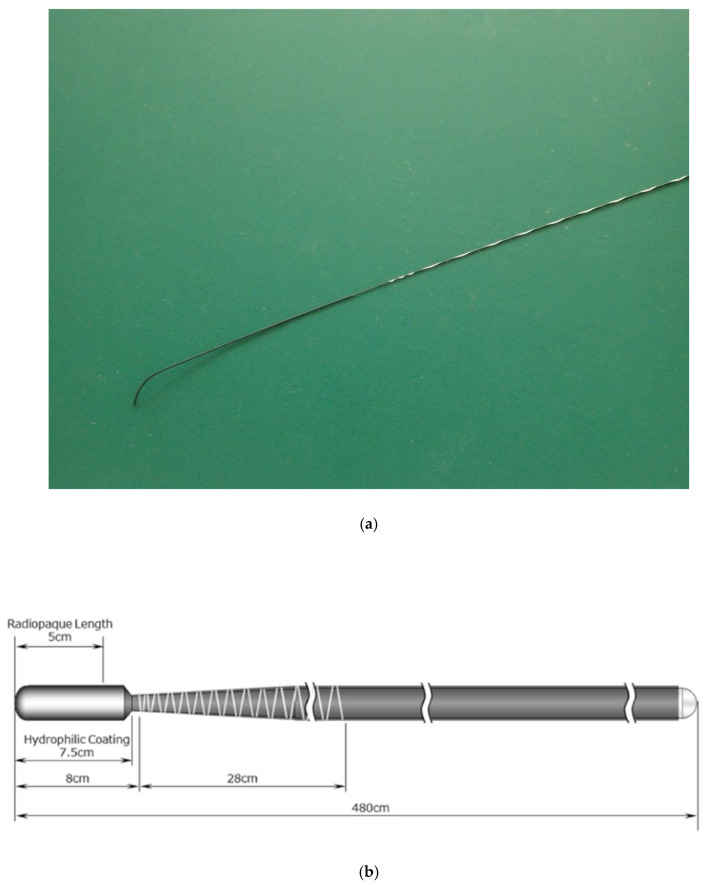
Properties of the M-Through guidewire (GW; Asahi Intecc Corp., Aichi, Japan). (**a**,**b**) The M-Through has a highly flexible 75 mm hydrophilic coated tip with a 50 mm (length) radiopaque jacket (schema provided by Hirata Company, Osaka, Japan).

Endoscopic treatments were performed according to the standards of each institution. Initial biliary cannulation with a standard ERCP catheter or sphincterotome was performed in all procedures. At the institutions participating in this study, the catheter cannulation method is basically the first choice as the biliary cannulation. After catheter cannulation, contrast is injected to confirm the bile duct. In CBD stone cases, after cholangiography, EST (endoscopic sphincterotomy) is performed for patients who have not undergone EST, followed by balloon dilation. In cases in which EST was previously performed, only balloon dilation was basically added. For removing stones, a balloon catheter was used initially, if difficult, a basket or mechanical lithotriptor was used. For cases in which complete stone removal was not achieved, plastic stents were placed. In MBO cases, the stricture was identified with the cholangiography, and then seeking the targeted bile duct with M-Through. In the case required multiple stents, second GW for the second stent was not defined. Finally, plastic or metal stents were placed in the bile duct. In cholecystitis cases, after cholangiography, the direction of branching of the cystic duct was identified, and the cystic duct was searched with M-Through. In the unsuccessful cases with M-Through, GW was changed. The second GW was not defined, and preferred GW of each manipulator was used. For successful placement of GW in the gallbladder, a plastic stent was placed. Patients were basically sedated deeply using midazolam or propofol as sedative and pethidine as analgesic. All procedures used the M-Through, a novel GW consisting of a flexible angled tip with a hydrophilic coating and a stiff shaft with a polytetrafluoroethylene coating. The GW is 0.025 inches in diameter and 400 cm in length (see Figure 1). The flexible hydrophilic tip provides a 1:1 torque ratio, and the stiff shaft provides adequate support. In many institutions, especially academic centers, the endoscopist has an assistant who operates the GW. In this study, we categorized assistants who manipulated the GWs by experience level as follows: “A” (less than five years of experience), “B” (five years to less than 10 years of experience), and “C” (10 or more years of experience).

All subjects gave their informed consent for inclusion before they participated in the study. The study was conducted in accordance with the Declaration of Helsinki, and the protocol was approved by the Ethics Committee of Juntendo University (20–230).

### 2.4. Endpoints and Statistical Analysis

For CBD stone extraction and endoscopic drainage for distal and hilar MBO, the primary endpoint was the procedure’s success rate without GW exchange. For cholecystitis procedures, we also analyzed the overall success rate and the next step needed (GW exchange and/or GW manipulator exchange). The number of cholecystitis procedures was considered insufficient for analysis and the success rate analysis was considered exploratory.

We used our clinical experience to determine the acceptable rate of success without GW exchange for each procedure. We set the threshold at 90% for CBD stone extraction, 80% for endoscopic drainage for distal and hilar MBO, and 50% for endoscopic drainage for acute cholecystitis. Sample sizes were calculated to determine the number of patients needed to evaluate effectiveness. The number of patients required to meet our target confidence level (95%) and margin of error (<20%) was 40 patients for CBD stone extraction, 60 patients for endoscopic drainage for distal and hilar MBO, and 97 patients for endoscopic drainage for acute cholecystitis. We ultimately decided to collect data from at least 60 patients with CBD stones and 60 patients with MBO. The number of cholecystitis procedures was considered insufficient for analysis and the success rate analysis was considered exploratory. We collected data from patients with cholecystitis requiring endoscopic drainage during the same time period for an exploratory analysis.

### 2.5. Definitions

We defined the primary endpoint as the success rate of endoscopic procedures for CBD stones and MBO without changing the GW evaluated in this study (the M-Through). In CBD stone cases, the success of the procedure was defined as successful insertion of any device required for stone removal or a stent placement without GW exchange. In MBO cases, success of the procedure was defined as the success of seeking and stenting the targeted branch of the bile duct without GW exchange. The use of additional GWs was allowed if required. When managing hilar strictures that requires multiple bile duct drainage, multiple GWs were required for multiple stenting. Multiple GWs were also needed in biliary cannulation cases that required pancreatic duct GW placement. In these cases, M-through was used for the procedure required for each case (e.g., stone removal, seeking, and stent insertion) after successful biliary cannulation.The overall success of the procedure was defined as whether the purpose of the planned procedure was achieved, including the change from M-through to another GW.In patients with CBD stones, a successful procedure was one in which the stones were extracted or a stent was placed. For patients with MBO and cholecystitis, a successful procedure was defined as the placement of at least one stent or one nasobiliary drainage tube.

## 3. Results

We collected clinical data from patients after applying the inclusion criteria. None of the patients were excluded. Patient characteristics are summarized in Table 1. We included 170 patients in the study, comprising 107 males and 63 females with an average age of 72.9 ± 13.1 years. The sample included 80 patients with CBD stones, 60 with MBO, and 30 with cholecystitis. Some patients with MBO had hilar and distal strictures. Details of the strictures are also summarized in Table 1.

Procedure-related outcomes are summarized in Table 2. The primary endpoint (success rate without GW exchange) was met in 100% of CBD stone procedures and 83.3% of MBO procedures. These rates cleared the thresholds that we had established prior to analysis. For cholecystitis cases, the primary endpoint was achieved in 43.3% of cases. This was slightly lower than our threshold. The overall success rates were 100% for CBD stone procedures, 96.7% for MBO procedures, and 80% for cholecystitis procedures.

**Figure 2:**
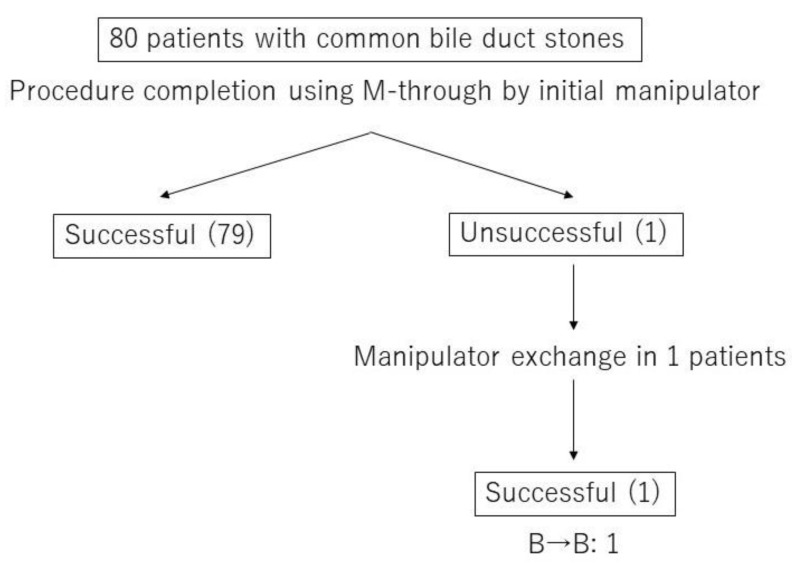
Flowchart of the procedural details including GW and/or manipulator exchange in common bile duct (CBD) stone removal cases.Experience level of GW manipulator; B was defined as five years or more but less than 10 years. One case succeeded by manipulator exchange from the experience level B to the experience level B.

Figure 2 presents a flowchart with details of the CBD stone procedures, including GW and/or manipulator exchanges. All CBD stone procedures were successful and completed without exchanging the GW. In one case, a senior doctor took over as the GW manipulator because of a difficult cannulation step.

**Figure 3:**
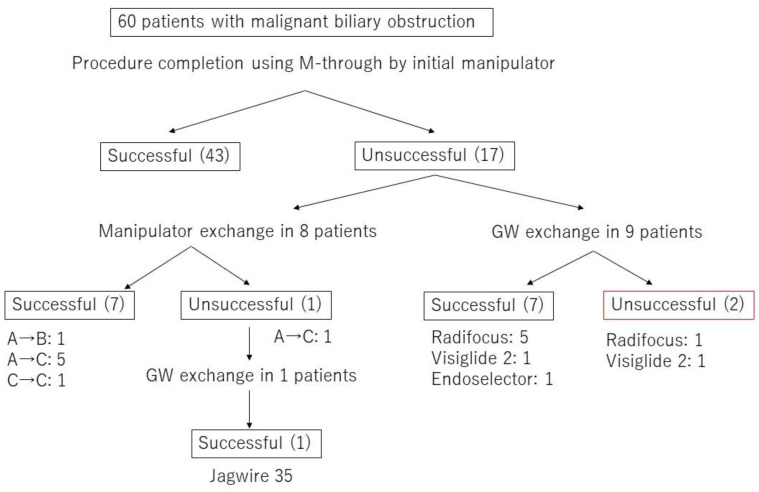
Flowchart of the procedural details including GW and/or manipulator exchange in malignant biliary obstruction (MBO) cases.Experience level of GW manipulator; A was defined as less than five years of experience, B as five years or more but less than 10 years, and C as 10 years or more.

Figure 3 presents a flowchart with details of the MBO procedures, including GW and/or manipulator exchanges. Forty-three MBO procedures were completed with the original GW and the original GW manipulator. Seven of the remaining 17 cases were completed with the original GW and a change in GW manipulator. This yielded an 83.8% rate of success without GW exchange (50/60 procedures). In the 17 procedures not completed with the original GW or the original GW manipulator, the GW was exchanged in 10 cases. The salvage GWs used in these cases were as follows: Radifocus (Terumo Corporation; 6 cases), Visiglide2 (Olympus Corp., Tokyo, Japan; 2 cases), EndoSelector (Boston Scientific Corporation; 1 case), and Jagwire (Boston Scientific Corporation; 1 case). In seven of these 10 cases, the GW was passed through the stricture successfully. In unsuccessful MBO cases with the original GW manipulator, 7 of 8 cases succeeded in the manipulator exchange. Including 1 case who changed GW after the manipulator exchange, 8 of 10 cases succeeded in changing GW. There was no significant difference in the success rate between the manipulator exchange and GW exchange (*p* = 1.00). To summarize these results, the 83.3% clinical success of MBO was brought to 96.7% when also allowing GW exchange, and the guidewire finally achieving canulation was a completely hydrophilic guidewire (6/10 60%) in the majority of cases.

**Figure 4:**
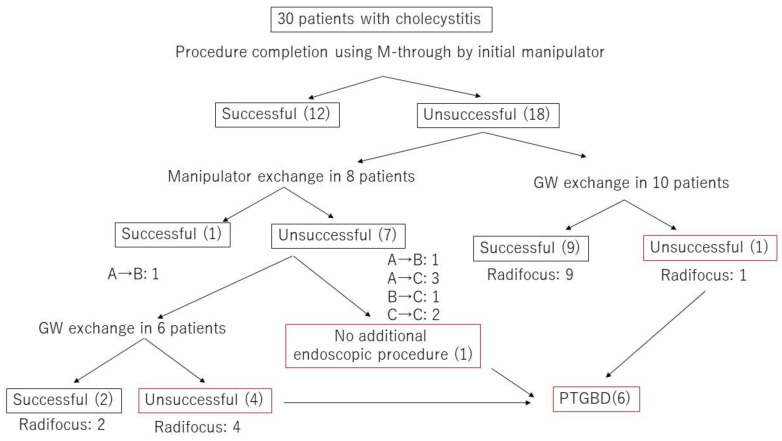
Flowchart of the procedural details including GW and/or manipulator exchange in cholecystitis cases.Experience level of GW manipulator; A was defined as less than five years of experience, B as five years or more but less than 10 years, and C as 10 years or more.

**Figure 5:**
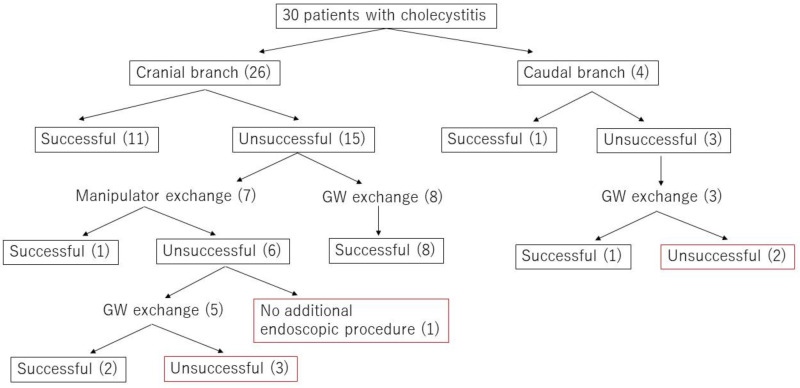
The cholecystitis cases were divided into two groups according to the direction of the cystic duct.

**Figure 6:**
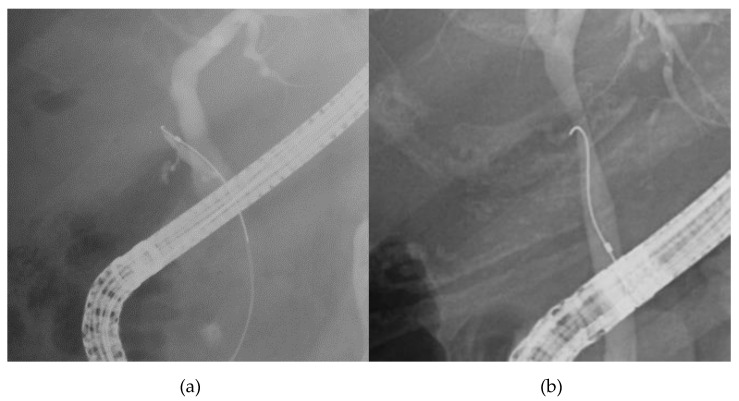
Cholangiography image of cranial branch and caudal branch; (**a**): cranial branch, (**b**): caudal branch.

Figure 4 presents a flowchart with details of the cholecystitis procedures, including GW and/or manipulator exchanges. Of the 30 cholecystitis procedures, 13 were completed with the original GW and either the original GW manipulator (12 cases) or a different GW manipulator (1 case). The success rate with M-Through was only 43.3%. Of the 17 procedures that required a GW change, 16 involved a switch to a Radifocus GW. In 68.8% of these procedures (11/16), the GW was passed through the cystic duct successfully and the procedure was completed. In the remaining six procedures, percutaneous transhepatic gallbladder drainage (PTGBD) was performed. To summarize these results, in unsuccessful cholecystitis cases with the original GW manipulator, 1 of 8 cases succeeded in the manipulator exchange. Including 6 cases who changed GW after the manipulator exchange, 11 of 16 cases succeeded in changing GW. There was significant difference in the success rate between the manipulator exchange and GW exchange (*p* = 0.03). We divided cholecystitis cases into two group according to the direction of the cystic duct. In 26 cases, the cystic duct branched toward the hepatic hilum side (cranial branch). In four cases, the cystic duct branched toward the papilla side (caudal branch) (see Figure 5 and Figure 6). In cases with cranial branches, the success rate of the procedure without GW exchange was 46.2% (12/26) and the success rate when the original GW was switched for a Radifocus GW was 76.9% (10/13). In cases with caudal branches, the success rate of the procedure without GW exchange was 25% (1/4) and the success rate when the original GW was switched for a Radifocus GW was 33.3% (1/3).

Table 3 shows the rates of successful device insertion and stent placement after biliary cannulation. In all CBD stone removal procedures, the insertion of devices for removing stones was completed without changing the GW. Placement of stents, including metallic stents, was possible in all procedures (regardless of type) except one. The unsuccessful procedure was a hilar MBO case in which it was difficult to insert a plastic stent due to a severe hilar stricture. We placed the stent successfully after switching to a 0.035 inch GW with high rigidity.

This study included some difficult biliary cannulation cases that required insertion of the M-Through into the pancreatic duct. Twelve cases required the pancreatic GW technique and four cases required the transpancreatic precut sphincterotomy technique. However, we did not observe any intraoperative GW-related adverse events such as perforation and bleeding due to manipulation.

## 4. Discussion

The world has recently seen advances in pancreatobiliary endoscopy. GW quality is a critical factor contributing to the effectiveness and efficiency of pancreatobiliary endoscopy. GWs have many uses: guiding catheters, supporting device insertion, exchanging devices, entering the branches of a duct, and passing through strictures. GWs vary in diameter (mainly 0.018, 0.025, and 0.035 inches), tip shape (straight or angled), and tip and shaft stiffness. The 0.035 inch GW used to be the preferred GW because of its stiffness, but there has been an increase in the use of 0.025 inch GWs since they have been produced with stiffer shafts. In the present study, we evaluated a new multipurpose 0.025 inch endoscopic GW: the M-Through. Because the functionality required of a GW depends on the procedure, we evaluated the M-Through in three procedures: CBD stone extraction, endoscopic drainage for distal and hilar MBO, and endoscopic drainage for acute cholecystitis.

For each procedure, we identified an acceptable rate of success without GW exchange. We set the threshold at 90% for CBD stone extraction, 80% for endoscopic drainage for MBO, and 50% for endoscopic drainage for cholecystitis. The rate of success without GW exchange was 100% for CBD stone removal procedures and 83.3% for MBO procedures. These rates exceeded the previously agreed upon thresholds. The rate of success without GW exchange for cholecystitis procedures was 43.3%, which was slightly below the acceptable threshold. To interpret these results, we should consider two aspects of GW functionality: the provision of rigidity to provide support and allow for device exchange and stent insertion, and the ability to pass through strictures and enter branches. Table 3 summarizes the success rates for device insertion and stent placement according to GW rigidity. In all CBD stone extractions, the devices for removing stones were inserted without changing the GW. Placement of stents, including metallic stents, was possible in every case but one. These results suggest that the GW’s rigidity is sufficient for device insertion and stent placement in most situations.

We evaluated the GW’s ability to pass through strictures in MBO procedures and the cystic duct in cholecystitis procedures. Forty-three of the MBO procedures were completed with the original GW and its original manipulator. Of the 17 unsuccessful procedures, eight involved manipulator changes. In 87.5% of these cases (7/8), the GW was then advanced through the stricture successfully. This result suggests that an 83.3% success rate (50/60) in passing through strictures can be achieved when the GW is manipulated with a certain level of skill. Of the 10 cases requiring GW exchange, one required exchange because the GW had insufficient rigidity for stenting and the remaining nine cases required GW exchange for passing through strictures. In seven of nine cases (77.8%), the GW was passed through the stricture after it was switched out. The hydrophilic GW was most commonly chosen. For endoscopic drainage for MBO, there is a high probability that the procedure can be completed using only the M-Through. If it is not possible to pass the GW through the strictures, the M-Through should be replaced with a hydrophilic GW. Subsequent stent placement can be done with the M-Through.

Endoscopic transpapillary gallbladder drainage has been reported to be effective, but the success rate varies from 50% to 100% because of difficulties in establishing a passage through ducts [10,11,12,13]. Of the 30 cholecystitis procedures, 12 were completed with the original GW and the original manipulator. Of the remaining 18, the manipulator was changed in eight cases. Only 12.5% of these (1/8) were successful. Sixteen cases (excluding one case in which PTGBD was performed without GW exchange) required the GW to be exchanged for a hydrophilic GW. In 11 of the 16 cases, the GW manipulator was then successful in passing the GW through the cystic duct. The average length of the procedure was approximately 1 h, which was longer than the average length of CBD stone removal and MBO procedures. Most of this hour was consumed with manipulating the GW in the cystic duct with X-ray guidance. This result suggests that the M-Through cannot be manipulated effectively in the cystic duct, and that a hydrophilic GW should be used from the beginning to reduce procedural time and exposure to X-rays.

There were no adverse events related to GW manipulation in this study. The M-Through consists of a soft tip with a hydrophilic coating and a shaft with high rigidity. The use of this type of GW has increased recently, and GW-related adverse events (e.g., perforation) have been reported [14,15,16,17]. The high rigidity of these GWs is good for providing support during device insertion but has the disadvantage of increasing the risk of perforation. Therefore, we should manipulate this type of GW carefully.

The retrospective nature of this study was a major limitation, as was the lack of a control group. In addition, we did not calculate or compare procedural costs. In conclusion, the 0.025 inch M-Through GW is a safe first choice for ERCP-related procedures. We achieved high rates of procedural completion without GW exchange. The M-Through can be used as a multipurpose GW, except in endoscopic drainage for acute cholecystitis. A prospective randomized controlled study comparing M-through with the other GWs is needed to establish the efficacy of M-through.

## Figures and Tables

**Table 1:** Patient characteristics.

	Common Bile Duct Stone (*n* = 80)	Malignat Biliary Obstruction (*n* = 60)	Cholecystitis (*n* = 30)	Total (*n* = 170)
Age (mean ± SD)	73.5 ± 14.2	72.4 ± 12.6	72.4 ± 11.3	72.9 ± 13.1
Sex (male: female)	54:26	37:23	16:14	107:63
Disease				
Common bile duct stone	80	–	–	80
Malignant biliary obstruction (Hilar/Distal)	–	60 (18/42)	–	60 (18/42)
Pancreatic cancer	–	20	–	20
Cholangiocarcinoma	–	21	–	21
Gallbladder cancer	–	1	–	1
periampullary carcinoma	–	5	–	5
Hepatocellular carcinoma	–	5	–	5
Metastatic biliary obstruction	–	8	–	8
Cholecystitis	–	–	30	30
Bismuth type (Ⅰ/Ⅱ/Ⅲa/Ⅲb/Ⅳ)	–	2/5/4/1/6	–	2/5/4/1/6
Prior papilla treatment				
Post EST	26	3	9	30
None	54	57	21	140
Level of initial GW manipulator *				
A	20 (25%)	19 (31.7%)	10 (33.3%)	49 (28.8%)
B	25 (31.3%)	18 (30%)	12 (40%)	55 (32.4%)
C	35 (43.8%)	23 (38.3%)	8 (26.7%)	66 (38.8%)

* Level of initial GW manipulator; A was defined as less than five years of experience, B as five years or more but less than 10 years, and C as 10 years or more. GW: guidewire.

**Table 2:** Details of procedure outcomes.

	Common Bile Duct Stone (*n* = 80)	Malignat Biliary Obstruction (*n* = 60)	Cholecystitis (*n* = 30)	Total (*n* = 170)
Overall success rate of procedure *n* (%)	80 (100%)	58 (96.7%)	24 (80.0%)	162 (95.3%)
Success rate of the procedure without GW exchange *n* (%)	80 (100%)	50 (83.3%)	13 (43.3%)	141 (82.9%)
Success rate of the procedure without GW and manipulator exchange *n* (%)	79 (98.8%)	43 (71.7%)	12 (40.0%)	141 (82.9%)
Succeeded by				
only manipulator exchange (using initial GW)	1 (1.3%)	7 (11.7%)	1 (3.3%)	9 (5.3%)
only GW exchange	0	7 (11.7%)	9 (30.0%)	16 (9.4%)
GW and manipulator exchange	0	1 (1.7%)	2 (6.7%)	3 (1.8%)
Procedure time (min)	23.7 ± 11.6	41.7 ± 23.7	53.4 ± 25.1	35.3 ± 22.4
Adverse events associated with GW	0	0	0	0

**Table 3:** Successful rate of device insertion and stent placement.

	Common Bile Duct Stone (*n* = 80)	Malignant Biliary Obstruction (*n* = 60)	Cholecystitis (*n* = 30)	Total (*n* = 170)
Device insertion				
Extraction balloon	100% (65/65)	—	—	100% (65/65)
Basket	100% (17/17)	—	—	100% (17/17)
ML	100% (17/17)	—	—	100% (17/17)
Brush catheter	—	100% (22/22)	—	100% (22/22)
Stent placement				
ENBD	100% (27/27)	100% (6/6)	—	100% (33/33)
ENGBD	—	—	100% (12/12)	100% (12/12)
Plastic stent	100% (9/9)	97.2% (35/36)	100% (12/12)	98.2% (57/58)
Metallic stent	—	100% (14/14)	—	100% (14/14)

ML, Mechanical lithotripter. ENBD, Endoscopic nasobiliary drainage. ENGBD, Endoscopic nasobiliary gallbladder drainage.
